# Presence of Equine and Bovine Coronaviruses, Endoparasites, and Bacteria in Fecal Samples of Horses with Colic

**DOI:** 10.3390/pathogens12081043

**Published:** 2023-08-15

**Authors:** Moritz Stummer, Vicky Frisch, Frauke Glitz, Barbara Hinney, Joachim Spergser, Jürgen Krücken, Irina Diekmann, Katharina Dimmel, Christiane Riedel, Jessika-Maximiliane V. Cavalleri, Till Rümenapf, Anja Joachim, Manolis Lyrakis, Angelika Auer

**Affiliations:** 1 Institute of Virology, University of Veterinary Medicine, 1210 Vienna, Austriakatharina.dimmel@vetmeduni.ac.at (K.D.); till.ruemenapf@vetmeduni.ac.at (T.R.); 2 Clinical Unit of Equine Internal Medicine, University of Veterinary Medicine, 1210 Vienna, Austria; vicky.frisch@vetmeduni.ac.at (V.F.); jessika.cavalleri@vetmeduni.ac.at (J.-M.V.C.); 3 Animal Clinic Würflach, 2732 Würflach, Austria; office@tierklinik-wuerflach.at; 4 Institute of Parasitology, University of Veterinary Medicine, 1210 Vienna, Austria; barbara.hinney@vetmeduni.ac.at (B.H.); anja.joachim@vetmeduni.ac.at (A.J.); 5 Institute of Microbiology, University of Veterinary Medicine, 1210 Vienna, Austria; joachim.spergser@vetmeduni.ac.at; 6 Institute for Parasitology and Tropical Veterinary Medicine, Freie Universität Berlin, 14163 Berlin, Germany; juergen.kruecken@fu-berlin.de (J.K.); irina.diekmann@fu-berlin.de (I.D.); 7 CIRI-Centre International de Recherche en Infectiologie, Univ Lyon, Université Claude Bernard Lyon 1, Inserm, U1111, CNRS, UMR5308, ENS Lyon, 46 allée d’Italie, 69364 Lyon, France; christiane.riedel@ens-lyon.fr; 8 Platform for Bioinformatics and Biostatistics, Department of Biomedical Sciences, University of Veterinary Medicine, 1210 Vienna, Austria; emmanouil.lyrakis@vetmeduni.ac.at

**Keywords:** equine coronavirus, horse, colic, bovine coronavirus, betacoronavirus, Austria

## Abstract

Acute abdominal pain (colic) is one of the major equine health threats worldwide and often necessitates intensive veterinary medical care and surgical intervention. Equine coronavirus (ECoV) infections can cause colic in horses but are rarely considered as a differential diagnosis. To determine the frequency of otherwise undetected ECoV infections in horses with acute colic, fresh fecal samples of 105 horses with acute colic and 36 healthy control horses were screened for viruses belonging to the *Betacoronavirus 1* species by RT-PCR as well as for gastrointestinal helminths and bacteria commonly associated with colic. Horses with colic excreted significantly fewer strongyle eggs than horses without colic. The prevalence of anaerobic, spore-forming, gram-positive bacteria (*Clostridium perfringens* and *Clostridioides difficile*) was significantly higher in the feces of horses with colic. Six horses with colic (5.7%) and one horse from the control group (2.8%) tested positive for Betacoronaviruses. Coronavirus-positive samples were sequenced to classify the virus by molecular phylogeny (N gene). Interestingly, in three out of six coronavirus-positive horses with colic, sequences closely related to bovine coronaviruses (BCoV) were found. The pathogenic potential of BCoV in horses remains unclear and warrants further investigation.

## 1. Introduction

Equine colic is defined as acute abdominal pain and represents a major health and welfare concern in horses worldwide. It is the most common reason for emergency veterinary treatment, often leading to critical conditions, and about 18% of horses with colic have to be euthanized or die [1,2,3]. In equine practice, determining the initial cause of colic is often challenging and inconclusive. Although risk factors such as changes in feeding, housing, or stress are known, there is limited evidence regarding virus infections as a cause of colic in horses [4].

Equine coronaviruses (ECoV) can cause severe febrile and enteric disease in adult horses [5,6,7]. Similar to severe acute respiratory syndrome (SARS) and Middle Eastern respiratory syndrome (MERS) coronaviruses, it belongs to the family *Coronaviridae*, genus *Betacoronavirus*. Equine coronavirus is assigned to the species *Betacoronavirus 1*, together with bovine coronavirus (BCoV), canine respiratory coronavirus (CRCoV), and human coronavirus (HCoV) OC43 [8]. The ECoV positive-sense, single-stranded RNA genome is 31 kb long [9]. The strain NC99 was the first ECoV isolated in cell culture from a diarrheic foal in North Carolina, USA, in 1999 [10] and its full-length genome was published by Zhang et al. [9]. To date, ECoV has only been detected in horses and donkeys [11]. Equine coronavirus infections can cause outbreaks at the farm level, affecting a large proportion of horses in a herd. Cases and outbreaks have been reported in the USA and Japan, as well as several European countries [5,6,7,12,13,14]. Clinical signs—including pyrexia, anorexia, and lethargy—are mostly self-limiting. However, severe clinical signs such as diarrhea, colic, and even death have been associated with ECoV infections [13,14,15,16,17,18,19,20,21].

Infectious agents most commonly associated with colic signs in horses are certain endoparasites and bacteria. The most common endoparasites of horses are the Cyathostominae (small strongyles), which normally lead to asymptomatic infections. However, in the rare event of larval cyathostominosis, they may cause colitis [22,23,24]. By contrast, large strongyles (Strongylinae) are rarely detected in Austria but are more pathogenic and often associated with colic signs. The most common and most pathogenic large strongyle is *Strongylus vulgaris*, the causative agent of verminous arteritis. Colic is induced by thromboembolic alterations in mesenteric vessels, leading to high mortality rates [25]. Other strongyles that might also cause colic signs are *Strongylus equinus* and *Strongylus edentatus* [26]. *Parascaris* spp. are known to cause enteritis or small intestinal obstruction especially in foals and young equids [25]. Due to the development of an adaptive immune response, heavy infections are rarely seen in adult horses [25,26,27,28]. *Anoplocephala perfoliata* can also impact GI-tract health. This tapeworm causes ileocecal invagination, intraluminal obstruction, and mucosal lesions or ulcers [25,29,30]. To date, the prevalence on German horse farms is estimated to be 1% as determined by copromicroscopy [24]; however, the seroprevalence in horses in Brandenburg was considerably higher at 16,2% [31]. There is no current information on nationwide parasite prevalence in Austria.

Opportunistic bacteria most commonly associated with enteric disease in horses are *Clostridioides (Cs.) difficile*, *Clostridium (Cl.) perfringens*, *Salmonella enterica*, and *Lawsonia intracellularis*. Infections with these pathogens can induce colitis that results in diarrhea, lethargy, anorexia, fever, and colic [32,33,34,35]. A decrease in microbial richness and diversity in the GI-tract of horses with colic has also been observed, while the importance of clostridia as a trigger of enterocolitis is discussed controversially [36,37,38]. Manship et al. (2019) compared the clinical features of enteric salmonellosis with those of ECoV infections and found them to be similar [39]. The authors suggest that in horses with fever and enteric clinical signs, both pathogens should be considered as causative agents [39]. In addition, *Salmonella enterica* subsp. *enterica* serovar Typhimurium (*Salmonella* Typhimurium) was isolated from 9–13% of horses displaying acute signs of colic [40].

In an outbreak setting, around one quarter of ECoV diseased horses develop colic signs [14,17]. Yet, ECoV infections—particularly on small farms—often remain undiagnosed, making it difficult to assess the pathogenetic role of ECoV infection in horses presenting with acute colic. To determine the pathogenic importance of ECoV in acute colic, we investigated the frequency of ECoV shedding in horses with acute colic, independent of the infection history, whilst simultaneously assessing fecal samples for parasites and bacteria potentially involved in colic. 

## 2. Materials and Methods

### 2.1. Animal Handling and Sampling

This study included 105 equine patients with acute signs of colic (colic patients; group P). In this study, acute colic was defined as the presence of acute colic signs for no longer than 72 h prior to sampling. Only horses with a minimum age of 6 months (median = 15 years, maximum = 33 years) were included in this study. Study horses were hospitalized at the animal clinic Wuerflach in Lower Austria (n = 30), at the University Equine Hospital, University of Veterinary Medicine, Vienna (n = 38), or were attended to by the veterinary ambulatory practice of the animal clinic Wuerflach (n = 37) between October 2021 and March 2022. Fecal samples were collected during the initial rectal examination of colic patients and stored at 4 °C. They were further processed in the respective laboratories within 3 days post-sampling. Thirty-six horses (three per month of study duration and clinic; convenience sampling) without evidence of internal medical abnormalities served as the control group (group C). They received treatment for ophthalmologic (27%) or orthopedic (27%) conditions, underwent surgical intervention emergencies (25%), sarcoid resection (5%), castration (11%), or served as companions for other patients (2%). Fecal samples of the control horses were collected immediately after defecation and stored at 4 °C as described above. 

Each sample was labeled with its designated group (colic patient: P or control horse: C) and a consecutive number. Written informed consent was obtained from all horse owners participating in this study. Owners were also requested to complete a comprehensive questionnaire regarding patient husbandry, feeding practices, usage, prior medical history, and any previous administration of antibiotics or anthelmintics. In addition, age, breed, and sex were recorded.

### 2.2. Clinical Examination

The general behavior, dietary condition, pulse rate, respiratory rate, capillary-refill time, mucosal color, skin turgor, and rectal temperature as well as the character of the peristaltic sounds from the patient´s group were assessed and documented during clinical examination. For the equine colic patients, findings concerning fecal consistency (normal or liquefied), rectal examination, and/or abdominal ultrasonography were used to make the diagnosis. The severity of colic was subjectively assessed by the examining veterinarian based on clinical findings and classified as mild, moderate, or severe. 

### 2.3. Virological Examination—Molecular Investigation for Betacoronaviruses

For viral RNA extraction, a fecal suspension (1%, in sterile PBS) was prepared, vortexed for 10 s, and centrifuged at 13,000 rpm for one minute. By employing the QIAamp 96 Virus QIAcube HT Kit using QIAcube HT (Qiagen, Hilden, Germany) according to the manufacturer’s instructions, 200 µL of supernatant was extracted. If not immediately processed, nucleic acid extracts were stored at −80 °C. 

An RT-qPCR assay targeting the polymerase gene of betacoronaviruses of veterinary importance was used for sample screening. Originally described for the detection of canine respiratory coronavirus [41], the assay was also validated for the detection of equine and bovine coronavirus-specific nucleic acids. All samples positive in this assay were additionally analyzed by an ECoV-specific RT-qPCR targeting the N gene, which was published by Pusterla and colleagues [7]. Ten-fold dilution series of defined DNA plasmid standards were tested side by side with the samples for absolute quantification. Real-time RT-qPCRs were performed using the Luna® Universal Probe One-Step RT-qPCR Kit (New England Biolabs) in a Rotor-Gene Q 5-plex machine (Qiagen, Hilden, Germany). Sample-free extracts (blanks) and no-template controls (NTC) served as negative controls in each PCR run. A 16S rRNA RT-qPCR was performed for each sample extract to control for the presence of PCR-inhibiting substances [42]. For all betacoronavirus-positive samples, the complete N coding sequence was determined. For this purpose, cDNA was generated with the LunaScript RT Master Mix Kit with an Oligo d(T)23 VN Primer (both New England Biolabs). Next, 2 µL of cDNA was used for complete N gene amplification employing the 2× Phanta Max Master Mix (Vazyme Biotech Co., Ltd., Nanjing, China) following the manufacturer´s instructions. Primer and probe sequences used for (RT-q)PCRs and sequencing are shown in Table 1. PCR products were cleaned with PCR Kleen Spin Columns (Bio-Rad Laboratories Ges.m.b.H., Vienna, Austria) and sent to Eurofins Genomics AT GmbH for sequencing. Obtained nucleotide sequences were analyzed, and a phylogenetic tree was generated (maximum likelihood method and Tamura-Nei model [43]) using MEGA version 11 [44]. 

### 2.4. Parasitological Examination—Fecal Parasitology and Molecular Investigation

Fecal samples were examined with a combined sedimentation/flotation method. Briefly, 20 g of feces was mixed with 250 mL of water, filtered into a beaker, and left to sediment overnight at 4 °C. The supernatant was discarded, and the sediment was centrifuged for 8 min at 690× *g*. The supernatant was discarded again, and the sediment was mixed with saturated sucrose solution (specific gravity 1.26) and centrifuged for 8 min at 690× *g* for flotation. Material from the surface of the solution was transferred with a wire loop to a glass slide and examined at 100× magnification under a light microscope. The egg shedding intensity was categorized based on the eggs counted per slide as follows: negative: 0; low: 1–9; moderate: 10–20; high: ≥ 21 eggs/slide. For statistical evaluation, samples with no or low-grade shedding were graded with zero (0), while those with moderate or high-grade egg count were graded with one (1). Samples of individual animals showing high levels of strongyle egg counts were subjected to larval culture for differentiation between cyathostomins and strongylins. Genomic DNA from the larvae was extracted using the NucleoSpin® Soil Kit (Macherey-Nagel). DNA was eluted with 50 μL of elution buffer. *Strongylus vulgaris* positive samples were identified using a specific qPCR [45] modified by Gehlen et al. [24] targeting a partial fragment of the internal transcribed spacer 2 (ITS-2) region. A second qPCR, amplifying a partial ITS-2 fragment of the species *Strongylus asini*, *S. edentatus*, and *S. equinus,* followed by a high-resolution melt analysis of amplification products as recently described [46] was also employed. PCR reactions contained 500 nM of each primer and 5 μL template DNA in 20 μL 1× GoTaq® qPCR Master Mix. To avoid PCR inhibition, the samples were used undiluted and diluted 1:5. Plasmid DNA representing all three species was used as a positive control and reference for the melting curve shapes (500, 50, and 5 copies per reaction in duplicates).

### 2.5. Bacteriological Examination—Bacterial Culture and Classification Procedures

For the bacteriological examination, swabs taken from the feces samples (approximately 100 mg) were plated onto Columbia agar III with 5 % sheep blood (general purpose medium for the isolation of non-fastidious and fastidious bacteria including anaerobes), CNA (Colistin-Nalidixic Acid) agar with 5% sheep blood, improved II (selective medium for the isolation of gram-positive bacteria), MacConkey II agar (selective medium for the isolation and differentiation of *Enterobacteriaceae* and other gram-negative bacteria), XLD (Xylose-Lysine-Desoxycholate) agar (selective medium for the isolation and differentiation of gram-negative enteric bacteria), *Campylobacter* blood-free agar, *Cs. difficile* agar with 7% sheep blood, and Sabouraud agar with Gentamicin and Chloramphenicol (selective medium for the isolation of yeasts and molds) (all BD Diagnostics, Vienna, Austria), using the three-phase streaking method. Plates were incubated aerobically at 37 °C (Columbia agar, CNA agar, MacConkey II agar, XLD agar), micro-aerobically at 42 °C (*Campylobacter* blood-free agar), or anaerobically at 37 °C (Columbia agar, *Cs. difficile* agar) for 48–72 h, and Sabouraud agar plates were incubated in ambient air at 28 °C for up to 7 days. Microbial growth was semi-quantitatively graded as light, moderate, or heavy depending on the occurrence and number of isolated colonies in streaking sections. For statistical evaluation, samples with no or low-grade shedding were graded with zero (0), while those with moderate and high-grade colony counts were graded with one (1). Colonies were identified at the species level by matrix-assisted laser desorption ionization—time of flight mass spectrometry (MALDI-TOF MS) as previously described [47]. For enrichment and selective isolation of *Salmonella*, swabs were incubated in Buffered Peptone Water (BD Diagnostics, Vienna, Austria) at 37 °C in ambient air for 24 h. After incubation, 100 µL of culture medium was transferred to Selenite and Rappaport-Vassiliadis R10 broth (both BD Diagnostics, Vienna, Austria), incubated at 42 °C for 24 h, and subsequently sub-cultured onto XLD agar, incubated aerobically at 37 °C for 24–48 h. Presumed *Salmonella* spp. colonies were identified by MALDI-TOF MS. Nested PCR was performed for the detection of *Lawsonia intracellularis* as described previously [48]. In total, 140 of the 141 samples were analyzed bacteriologically. One of the samples from the control group was lost in transit and not analyzed.

### 2.6. Statistical Analysis

The association between the incidence of colic and the presence of various infectious agents (betacoronaviruses, endoparasites, and bacteria) was evaluated via Fisher’s exact tests (FET) for count data in R (R version 4.1.2, function *fisher.test*) [49]. The reported odds ratios are translated as the ratio between the odds of colic occurrence in the presence and absence of the infectious agent, respectively. The resulting *p*-values were adjusted for multiple testing according to Benjamini and Hochberg’s false discovery rate (FDR) correction [50]. Significance was declared at an FDR cut-off of 5%.

## 3. Results

### 3.1. Clinical Findings

Based on the findings obtained from clinical and rectal examinations, as well as abdominal ultrasonography, the predominant pathological findings observed in horses with colic in this study were located in the ascending large colon (58.2%). These were followed by abnormal findings of the caecum (14.3%), the stomach (11.2%), and the small intestine (6.1%). Gastrointestinal abnormalities could not be identified in seven cases presenting with colic (6.7%) by the examination techniques named previously. In 39 cases (37.1%), the severity of colic was scored as moderate or high, and low for 66 cases (62.9%). In 21 cases (20.0%), the rectal temperature was reported as higher than 38.0 °C. Furthermore, 17 patients (16.2%) showed decreased fecal consistency during the colic episode. Five patients showed both of the latter clinical signs. 

### 3.2. Virological Findings—Molecular Investigation for Betacoronaviruses

**Figure 1:**
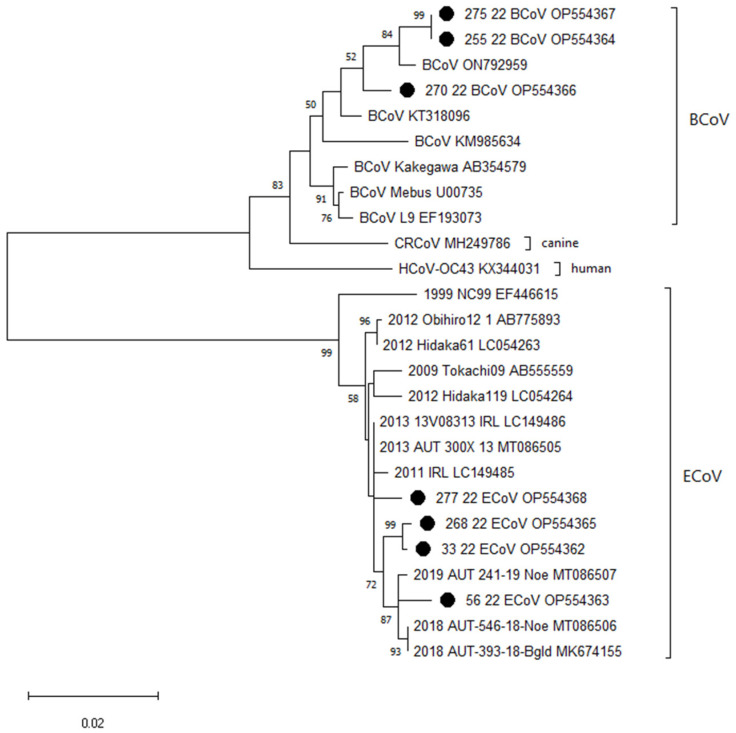
Phylogenetic analysis of complete N gene nucleotide sequences (ECoV: 1341 nucleotides, BCoV: 1347 nucleotides). Numbers next to the nodes represent the bootstrap values; only bootstrap values > 70% are shown (1000 replicates). The scale bar represents a length corresponding to 0.02 nucleotide substitutions per site. Sequences obtained in this study are marked with a black dot. Other sequences were downloaded from GenBank. Maximum Likelihood method and Tamura-Nei model [43] evolutionary analyses were conducted in MEGA11.

Betacoronavirus-specific nucleic acids were detectable by RT-qPCR in six out of one hundred and five colic patients (group P, 5.7%) and in one out of thirty-six horses from the control group (group C, 2.8%). Viral loads ranged from 1.8 × 10^6^ to 1.1 × 10^9^ genome equivalents (GE)/g feces. No association between betacoronavirus presence and colic occurrence was detected (FET odds ratio = 2.11, FDR = 0.932). ECoV-specific nucleic acids could be amplified by conventional RT-PCR in four of the betacoronavirus-positive samples (three from group P and the positive sample from group C). Subsequent sequence analyses of the complete N gene confirmed an ECoV infection in the same three horses from group P and one horse from group C. The sequences obtained from the three remaining betacoronavirus 1-positive samples were clustered with bovine coronaviruses (Table 2). A phylogenetic tree showing N gene sequences of equine and bovine coronaviruses is shown in Figure 1. The obtained sequences are registered in GenBank with the accession numbers: OP554362 (33/22), OP554363 (56/22), OP554364 (255/22), OP554365 (268/22), OP554366 (270/22), OP554367 (275/22), and OP554368 (277/22).

Among the horses in the colic group, six individuals tested positive for ECoV or BCoV. These horses exhibited mild to moderate colic signs and did not display a body temperature exceeding 38 °C during their initial clinical examination. Two of these horses had decreased fecal consistency. One patient showed lethargy and two presented with mild to moderately decreased body condition, including one showing both clinical signs. Four of these horses showed an impaction of the ascending colon. Two betacoronavirus-positive horses (patients 275/22 and 268/22) showed onset of fever 12 days after arrival at the clinic with inner body temperatures rising to 38.5 °C for one day; patient 275/22 showed a maximum of 39.4 °C intermittently over 14 days. Additionally, patient 268/22—initially presenting with mild signs of colic—showed increasingly severe colic during the course of the disease and surgical correction of nephrosplenic space entrapment of the large colon had to be performed. This case represents the only coronavirus-infected horse with colic that underwent surgical treatment. The ECoV-positive horse from the control group displayed no clinical signs related to an ECoV infection. Table 2 gives an overview of virological findings as well as some clinical, parasitological, and bacteriological results in the coronavirus-positive horses. 

### 3.3. Parasitological Findings—Fecal Parasitology and Molecular Investigation

Strongyle eggs were detected in 53 of the 141 samples (low to high grade, 37.6%) (Table 3) and showed a significant negative association with colic occurrence (FET odds ratio = 0.29, FDR = 0.025). Considering the coronavirus-infected horses, only one ECoV-positive horse showed a low intensity of strongyle egg shedding.

Furthermore, four (2.8%) and three (2.1%) of the one hundred forty-one samples were positive for *Parascaris* spp. and *A. perfoliata*, respectively. Two horses with *Parascaris* spp. were colic patients and coronavirus-infected, while two horses with *A. perfoliata* were colic patients but not coronavirus-infected. For both of these parasites, no significant association with colic occurrence was detected (*Parascaris* spp. FET odds ratio = 0.33, FDR = 0.423 and *A. perfoliata* FET odds ratio = 0.68, FDR = 1). One sample of one animal of the control group tested weakly positive for *S. vulgaris* by PCR; thus, small strongyles were predominant on all farms.

### 3.4. Bacteriological Findings—Bacterial Culture and Classification Procedures

Microbial species most frequently isolated from both groups were *Escherichia coli* (87.9%), *Enterococcus* spp. (81.4%), *Streptococcus equinus* (57.1%), *Bacillus* spp. (36.4%), *Cl*. *perfringens* (33.6%), and *Lactobacillus* spp. (32.9%). Most notable, anaerobic spore-forming gram-positive bacteria combined (*Cl. perfringens* and *Cs. difficile*) were significantly more common in the feces of horses with colic (FET odds ratio = 4.12, FDR = 0.01) (Table 4). From one control horse, *Salmonella* Infantis was isolated. All horses tested negative for *Campylobacter* spp. and *L. intracellularis*. 

## 4. Discussion 

Several risk factors (e.g., change in feeding, stress, parasitic and bacterial infections) have been associated with equine colic [3,4]. Nevertheless, the causative factor for an individual colic event often remains unknown. In our study, 105 horses with acute colic were examined clinically and their fecal samples were tested for the presence of parasites, bacteria, and betacoronaviruses. 

Out of the 105 horses included in the study, betacoronavirus DNA was detected in the fecal samples of seven horses, six of which were presented for colic. Four out of six betacoronavirus-infected horses with colic showed an impaction of the large colon; one of them further presented a left dorsal displacement of the colon. The other two coronavirus-infected horses showed no abnormal rectal findings. Alterations of the large colon are the predominant colic variant found in this study. Thus, a causative association of the BCoV or ECoV infection with the diagnosed colonic pathologies does not seem likely. Only a few studies described cases of ECoV infections in association with impactions including large cecal, large colon, and small colon impactions [19,51,52]. 

Two horses testing positive for ECoV showed liquefied fecal consistency and mild to moderate colic signs. None of them showed pyrexia during their colic episode. Two other betacoronavirus-infected horses showed elevated body temperature later in the course of the disease. Thus, only four out of the seven horses that tested positive for coronaviruses showed expectable clinical signs such as decreased fecal consistency, lethargy, or increased rectal temperature, which supports that clinical signs are rather nonspecific, as reviewed recently [53].

Parasites are a minor cause of colic signs [24,27]. In the present study, common endoparasites were also detectable in both the patients and the control animals. Horses with colic excreted significantly fewer strongylid eggs than horses without colic. One possible cause for this seemingly contradictory finding could be larval cyathostominosis. Here, the larvae erupt from the intestinal mucosa and thus cause colic; fecal sample examinations are then usually negative [22]. Furthermore, a negative influence of anthelmintics on the intestinal flora could be the reason for more frequent colic in the absence of parasites [54,55].

Bacterial pathogens such as *S. enterica, Cl. perfringens,* and *Cs. difficile* have been frequently associated with colitis in horses but may also be present in the intestinal tract of healthy individuals [35]. Shedding prevalence of these opportunistic pathogens can vary markedly with health status, season, and geographic location, ranging from 0.5–7%, 0–25%, and 0–41% for *S. enterica*, *Cs. difficile*, and *Cl. perfringens*, respectively [37,56,57]. In the present study, anaerobic, spore-forming, gram-positive bacteria (*Cl. perfringens* and *Cs. difficile*) were significantly more prevalent in the intestinal tract of horses with colic, underscoring their ability to overgrow and outcompete other microbiota when dysbiotic conditions occur [37,38]. 

Apart from fever and inappetence, colic is a frequent clinical sign observed in 19% of the cases during ECoV outbreaks [17]. However, in smaller herds, ECoV infections may remain undetected because the disease is often mild and self-limiting, and specific diagnosis is not part of routine procedures. In our study, we found a prevalence of *Betacoronavirus 1* of 5.7% in horses with colic and 2.8% in the control group. Consequently, no statistically significant difference in *Betacoronavirus 1* presence between colic-affected horses and control horses could be determined, indicating a minor role of *Betacoronavirus 1* in the pathogenesis of colic. In a study by Sanz et al. (2019) [58], only one out of 65 hospitalized horses with gastrointestinal signs (1.5%) tested positive for ECoV-specific RT-qPCR. In fecal samples obtained from an additional four gastrointestinal patients and five orthopedic patients in this report, intriguingly, the presence of CoV-like particles was detected through electron microscopy analysis [58]. In another study from 2013, BCoV-like sequences in two healthy horses could be found [59]. Our study supports this finding, since we were also able to detect BCoV in an animal species in which these viruses have not been previously described. In a review article by Zhu et al. from 2023 [60], the authors confirm that BCoV is of great significance in the field of cross-species transmission and has important biosafety implications. Thus, future long-term epidemiological surveys considering BCoVs/bovine-like coronaviruses from birds, cattle, other animals, humans, and also horses are required. 

We could detect ECoV nucleic acids in one control horse. This once more underlines the possible role of virus transmission by asymptomatic carriers as already described before [7,14,20]. Our study revealed that bovine coronaviruses could potentially be overlooked in ECoV-specific diagnostic assays. Thus, we recommend using a Pan-*Betacoronavirus 1* RT-qPCR for diagnosing coronaviruses in horses and combining it with an ECoV-specific assay or sequence analysis. In a coronavirus disease outbreak on an American miniature horse breeding farm in New York in 2013, samples were also investigated by a Pan-Betacoronavirus PCR but were not further differentiated [20]. 

Two of the BCoV N gene sequences obtained in this study are identical. Interestingly, the respective horses were not known to have had direct contact with each other, but they were treated at the same veterinary clinic at different time points. Cross contamination was avoided as much as possible, and negative controls and blanks were always negative, and repeated workup starting from the original sample on yielded reproducible results, making it unlikely that this finding is an artifact caused by contamination. None of the BCoV-infected horses were kept together with cattle, raising the question of the source of infection.

The ability of Betacoronaviruses to cross species barriers is well known and harbors the risk of outbreaks and pandemics within the human population. Aside from the SARS-CoV-2 pandemic and MERS-CoV outbreaks, which have had an impressive impact on human health in recent times, another spillover event threatened the world between 1889 and 1890. A pathogen causing respiratory signs, malaise, fever, and affection of the central nervous system quickly spread globally and killed around one million people [61]. There is evidence that this so-called ‘Russian flu’ was not caused by an influenza virus as previously assumed but by the emergence of human coronavirus OC43 (HCoV-OC43) after a BCoV spillover event from bovine livestock [62,63]. In addition, CRCoV, a respiratory pathogen of dogs, seemed to be the result of transmission from cattle [64,65]. Meanwhile, BCoV sequences were isolated from different wild ruminant species, camelids, and tapirs [66,67,68], but their role in horses and their possible clinical importance is unclear. While still struggling with the previous coronavirus pandemic, we should be aware of viruses crossing species barriers in our companion animals, too.

This study confirms the role of anaerobic, spore-forming, and gram-positive bacteria in equine colic and demonstrates that betacoronavirus 1 seems to play no major role as an etiological cause of colic in Austrian horses. Given the prevalence of 5% in all the horses included in this study, there is a non-neglectable risk of betacoronavirus 1 transmission especially in the context of veterinary hospitals and competitions. The role of BCoV in horses should be clarified in further studies, not only in terms of possible pathogenicity but also in terms of its possible zoonotic potential.

## Figures and Tables

**Table 1:** List of primer and probe sequences used in this study.

Primer/Probe Sequences	Sequence 5′-3′	Source
qBetaCoV1-F	ACGTGGTGTTCCTGTTGTTATAGG	[41]
qBetaCoV1-R	AACATCTTTAATAAGGCGACGTAACAT	
qBetaCoV1-P	FAM-CCACTAAGTTTTATGGCGGCTGGGATG-BHQ1	
qECoV-N-F	TGGGAACAGGCCCGC	[7]
qECoV-N-R	CCTAGTCGGAATAGCCTCATCAC	
qECoV-N-P	FAM-TGGGTCGCTAACAAG-BHQ1	
ECoV-29316-F	CAGGCATGGACACCGCATTG	[14]
ECoV-30730-R	CCAGGTGCCGACATAAGGTTCAT	[5]
BetaCoV-30000-R ^1^	CTTGATCCTGCACTAGAGGCTC	[14]
ECoV-30509-F ^1^	GATGATGGGACGAATATGAGC	This study
BCoV-30578-F ^1^	GTACACTTTCAGGTTTTGAGACC	

^1^ internal sequencing primers.

**Table 2:** Overview of the ECoV/BCoV-positive horses: Results of the molecular detection of coronaviruses in fecal samples, the first clinical examination, and the semiquantitative assessment of bacteria and parasites in feces. P: patient group with colic, C: control group; severity/abundance of colic and Strongyles: high (+++), moderate (++), low (+), absent (-); * later onset of pyrexia; ** tested positive for *P. equorum*.

Group	Horse ID	Virus and Viral Loads	Rectal Temp. [°C]	Fecal Consistency	Findings of Rectal Examination	Severity of Colic	Strongyles	Bacteria
P	33/22	ECoV 1.14 × 10^9^	38.0	Soft	No abnormal findings	++	-	+++ *E. coli* +++ *S. dysgalactiae* ssp. *equisimilis* ++ *Enterococcus faecalis* +++ *Candida* spp.
P	255/22	BCoV 3.47 × 10^7^	37.2	formed	Colonic impaction	+	-	++ *E. coli* ++ *Enterococcus faecalis* +++ *Cl. perfringens*
P	268/22	ECoV 1.87 × 10^9^	37.3 *	formed	Colonic impaction and left dorsal displacement of the ascending colon	+	- **	++ *E. coli* +++ *S. equinus* ++ *Enterococcus faecalis*
P	270/22	BCoV 1.77 × 10^6^	37.5	formed	No abnormal findings	+	+	++ *E. coli* ++ *Enterococcus faecalis* ++ *Cl. perfringens* +/++ *Cs. difficile*
P	275/22	BCoV 3.19 × 10^6^	37.5 *	formed	Colonic impaction	++	- **	++ *E. coli* +++ *S. equinus* ++ *Enterococcus faecium*
P	277/22	ECoV 2.57 × 10^8^	37.9	Soft, sticky	pelvic flexure impaction	+	-	+++ *Bacillus* spp. ++ *S. equinus* ++ *Enterococcus faecium*
C	56/22	ECoV 6.20 × 10^6^	Referred for an ophthalmologic consult. No clinical evidence for viral infection or gastrointestinal disease.				-	+ *E. coli* +++ *Enterococcus faecalis*

**Table 3:** Evidence of strongyle shedding during parasitological examinations.

Strongylid Egg Shedding	Patients	Controls	N Total
negative	71 (67.6%)	17 (47.2%)	88
low-grade	18 (17.1%)	5 (13.9%)	23
moderate	2 (1.9%)	6 (16.7%)	8
high-grade	14 (13.3%)	8 (22.2%)	22

**Table 4:** Bacteria identified in the fecal samples of investigated horses. *Cl. perfringens* and *Cs. difficile* were grouped together (as anaerobic spore-forming gram-positive bacteria) for Fisher’s exact test.

Organism	Family	Order	Patients	Controls	FET Odds Ratio	FDR
*E. coli*	*Enterobacteriaceae*	*Enterobacterales*	95 (90.5%)	28 (80%)	2.36	0.244
*S. equinus*	*Streptococcaceae*	*Lactobacillales*	66 (62.9%)	14 (40%)	2.52	0.107
*S. dysgalactiae*	*Streptococcaceae*	*Lactobacillales*	14 (13.3%)	4 (11.4%)	1.19	1
*Enterococcus* spp.	*Enterococcaceae*	*Lactobacillales*	86 (81.9%)	28 (80%)	1.13	0.984
*Bacillus* spp.	*Bacillaceae*	*Bacillales*	33 (31.4%)	18 (51.4%)	0.44	0.118
*Lactobacillus* spp.	*Lactobacillaceae*	*Lactobacillales*	30 (28.6%)	16 (45.7%)	0.48	0.21
*Cl. perfringens*	*Clostridiaceae*	*Clostridiales*	41 (39%)	6 (17.1%)	4.12	0.01
*Cs. difficile*	*Peptostreptococcaceae*	*Eubacteriales*	22 (21%)	2 (5.7%)		

## Data Availability

The sequences obtained in this study are publicly available in GenBank database and can be retrieved using the accession numbers OP554362 (33/22), OP554363 (56/22), OP554364 (255/22), OP554365 (268/22), OP554366 (270/22), OP554367 (275/22) and OP554368 (277/22).
